# LiDAR-based reference aboveground biomass maps for tropical forests of South Asia and Central Africa

**DOI:** 10.1038/s41597-024-03162-x

**Published:** 2024-04-04

**Authors:** Suraj Reddy Rodda, Rakesh Fararoda, Rajashekar Gopalakrishnan, Nidhi Jha, Maxime Réjou-Méchain, Pierre Couteron, Nicolas Barbier, Alonso Alfonso, Ousmane Bako, Patrick Bassama, Debabrata Behera, Pulcherie Bissiengou, Hervé Biyiha, Warren Y. Brockelman, Wirong Chanthorn, Prakash Chauhan, Vinay Kumar Dadhwal, Gilles Dauby, Vincent Deblauwe, Narcis Dongmo, Vincent Droissart, Selvaraj Jeyakumar, Chandra Shekar Jha, Narcisse G. Kandem, John Katembo, Ronald Kougue, Hugo Leblanc, Simon Lewis, Moses Libalah, Maya Manikandan, Olivier Martin-Ducup, Germain Mbock, Hervé Memiaghe, Gislain Mofack, Praveen Mutyala, Ayyappan Narayanan, Anuttara Nathalang, Gilbert Oum Ndjock, Fernandez Ngoula, Rama Rao Nidamanuri, Raphaël Pélissier, Sassan Saatchi, Le Bienfaiteur Sagang, Patrick Salla, Murielle Simo-Droissart, Thomas B. Smith, Bonaventure Sonké, Tariq Stevart, Danièle Tjomb, Donatien Zebaze, Lise Zemagho, Pierre Ploton

**Affiliations:** 1 Forestry and Ecology Group, National Remote Sensing Centre, ISRO, Hyderabad, 500 037 India; 2 Indian Institute of Space Science and Technology (IIST), Thiruvananthapuram, Kerala India; 3 College of Forestry, Oregon State University, Corvallis, OR 97331 USA; 4 AMAP, Univ Montpellier, IRD, CNRS, INRAE, CIRAD, Montpellier, France; 5 Center for Conservation and Sustainability, Smithsonian National Zoo and Conservation Biology Institute, Washington, DC USA; 6 Ecole Nationale des Eaux et Forêts de Mbalmayo, Ministère Des Forêts Et De La Faune, Mbalmayo, Cameroon; 7 Department of Ecology, French Institute of Pondicherry, Pondicherry, 605 001 India; 8 Institut de pharmacopée et de médecine traditionnelle (Herbier National du Gabon), CENAREST, Libreville, Gabon; 9 National Biobank of Thailand (NBT), National Science and Technology Development Agency, Klong Luang, Pathum Thani Thailand; 10 Department of Environmental Technology and Management, Faculty of Environment, Kasetsart University, Bangkok, 10900 Thailand; 11 National Institute of Advanced Studies (NIAS), Bangalore, India; 12 Plant Systematics and Ecology Laboratory, Higher Teachers’ Training College, University of Yaoundé I, P.O. Box 047, Yaoundé, Cameroun; 13 International Joint Laboratory DYCOFAC, IRD-UYI-IRGM, P.O Box 1857, Yaoundé, Cameroon; 14 International Institute of Tropical Agriculture (IITA), BP 2008 (Messa), Yaoundé, Cameroon; 15 Center for Tropical Research, Institute of the Environment and Sustainability, University of California Los Angeles, Los Angeles, CA 90095 USA; 16 Institut Supérieur d’Etudes Agronomiques de Bengamisa, République Démocratique du Congo, Congo, France; 17 Department of Geography, University College London (UCL), London, UK; 18 School of Geography, University of Leeds, Leeds, UK; 19 Ecologie des Forêts Méditerranéennes (URFM), INRAE, F-84914 Avignon, France; 20 Dja Wildlife Reserve, Ministry of Forestry and Wildlife, Yaoundé, Cameroon; 21 Jet Propulsion Laboratory, California Institute of Technology, Pasadena, CA 91109 USA; 22 Missouri Botanical Garden, Africa & Madagascar Program, 4344 Shaw Blvd., St. Louis, Missouri 63110 USA

**Keywords:** Forestry, Carbon capture and storage, Forestry

## Abstract

Accurate mapping and monitoring of tropical forests aboveground biomass (AGB) is crucial to design effective carbon emission reduction strategies and improving our understanding of Earth’s carbon cycle. However, existing large-scale maps of tropical forest AGB generated through combinations of Earth Observation (EO) and forest inventory data show markedly divergent estimates, even after accounting for reported uncertainties. To address this, a network of high-quality reference data is needed to calibrate and validate mapping algorithms. This study aims to generate reference AGB datasets using field inventory plots and airborne LiDAR data for eight sites in Central Africa and five sites in South Asia, two regions largely underrepresented in global reference AGB datasets. The study provides access to these reference AGB maps, including uncertainty maps, at 100 m and 40 m spatial resolutions covering a total LiDAR footprint of 1,11,650 ha [ranging from 150 to 40,000 ha at site level]. These maps serve as calibration/validation datasets to improve the accuracy and reliability of AGB mapping for current and upcoming EO missions (viz., GEDI, BIOMASS, and NISAR).

## Background & Summary

Tropical forests play a vital role in the Earth’s carbon cycle and contribute largely to uncertainties in the global carbon budget^1^. Methods to accurately map and monitor tropical forest carbon – or aboveground biomass (AGB) – are thus urgently needed to improve Earth system models and to help design carbon emission mitigation strategies in the context of Reducing Emissions from Deforestation and forest Degradation (REDD+)^2,3^. In the last decade, spaceborne Earth Observation (EO) data in combination with forest inventory measurements have been extensively used to generate spatially continuous AGB maps at pan-tropical scale using different modelling strategies^3–6^. However, existing broad-scale maps show divergent estimates among themselves and differ from field-derived forest AGB stocks at different spatial scales^1,4,5,7^, indicating the presence of high uncertainties in prediction maps. To improve the accuracy and reliability of AGB maps over the tropics, several ongoing and upcoming EO missions (NASA’s GEDI, ESA’s BIOMASS, NASA-ISRO’s NISAR and JAXA’s ALOS-4 missions, notably) have been specifically designed to collect satellite data sensitive to forest structure, hence to forest AGB^6,8–10^. While these new spaceborne datasets will undoubtedly revolutionise broad-scale forest AGB mapping, a network of high-quality reference data is needed to calibrate and validate the mapping algorithms^11,12^. Besides, using the same sets of reference data across different EO missions would vastly improve the comparability and confidence in the derived AGB maps, enabling their use in a wide range of science, policy, and management applications^13^.

Generating reference AGB observations over a given area is challenging since forest AGB is not directly measured through destructive sampling, but instead estimated from tree inventories and a series of statistical models propagating substantial uncertainties^5^. It is therefore required to reduce as far as possible and quantify the uncertainty on reference AGB predictions. In this context, the Committee on Earth Observation Satellite (CEOS) has established a good practice protocol for generating reference AGB dataset, to facilitate the production and warrant the consistency of next-generation biomass products^14^. The protocol suggests developing reference AGB maps over sizable areas using local forest sample plots and LiDAR data acquired using aerial platforms (hereafter airborne LiDAR). Airborne LiDAR data is currently the most informative data type for characterizing forest structure and deriving AGB maps at the landscape scale, provided they are adequately calibrated with respect to local environmental gradients and forest structural and species variability^15^. Besides, the high spatial resolution of airborne LiDAR data (or of derived AGB predictions) can easily be aggregated to coarser resolutions, thus bridging the scale gap between field data and the resolution of upcoming EO sensors (e.g. 100 m for NISAR, 200 m for BIOMASS).

The establishment and long-term maintenance of a network of reference forest AGB observatories across the tropics entails a myriad of challenges, particularly concerning the representativeness of the network^12^. Ideally, the network should be relatively evenly distributed in space and cover the main environmental gradients. While scientific discussions on site selection are on-going^12^, the Global Ecosystem Dynamics Investigation (GEDI) sensor on-board the International Space Station has already acquired data for a longer period than its initially projected lifetime. Data users would benefit from open-access reference AGB data, particularly in Asia where large geographic regions are not represented in the calibration/validation dataset of GEDI biomass mapping algorithm^16,17^. Besides the notion of spatial representativeness, hurdles related to the temporal mismatch between reference AGB and EO data should not be neglected. Rapid growth in regenerating forests or forest clearing/degradation – which notably characterise rural landscapes around central African cities, where slash-and-burn agriculture induces relatively fast dynamics – could rapidly make tens of thousands of GEDI data shots unusable. We argue that airborne LiDAR data acquired during GEDI lifetime over rapidly changing landscapes are invaluable and should be utilized to improve GEDI biomass mapping algorithms, notably on the lower-end of the forest biomass gradient to best capture forest degradation and regeneration gradients.

**Fig. 1:**
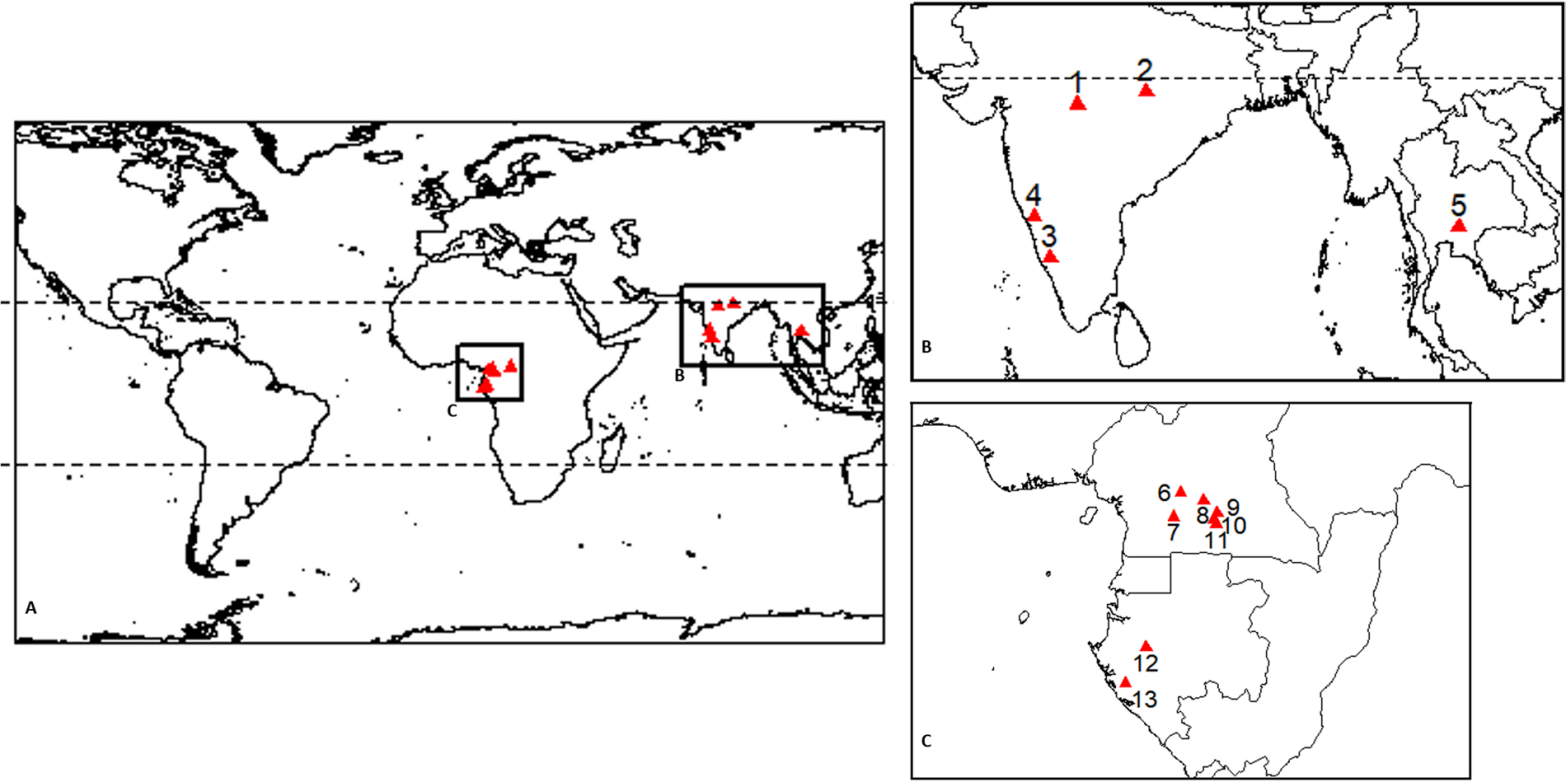
(**A**) Overview map showing the locations of sampling sites (n = 13) used in the current study. Outlined regions are expanded in (**B**): South Asian region and in (**C**): Central African region). Sampling site names and descriptions associated with site numbers are provided in Table 1.

**Fig. 2:**
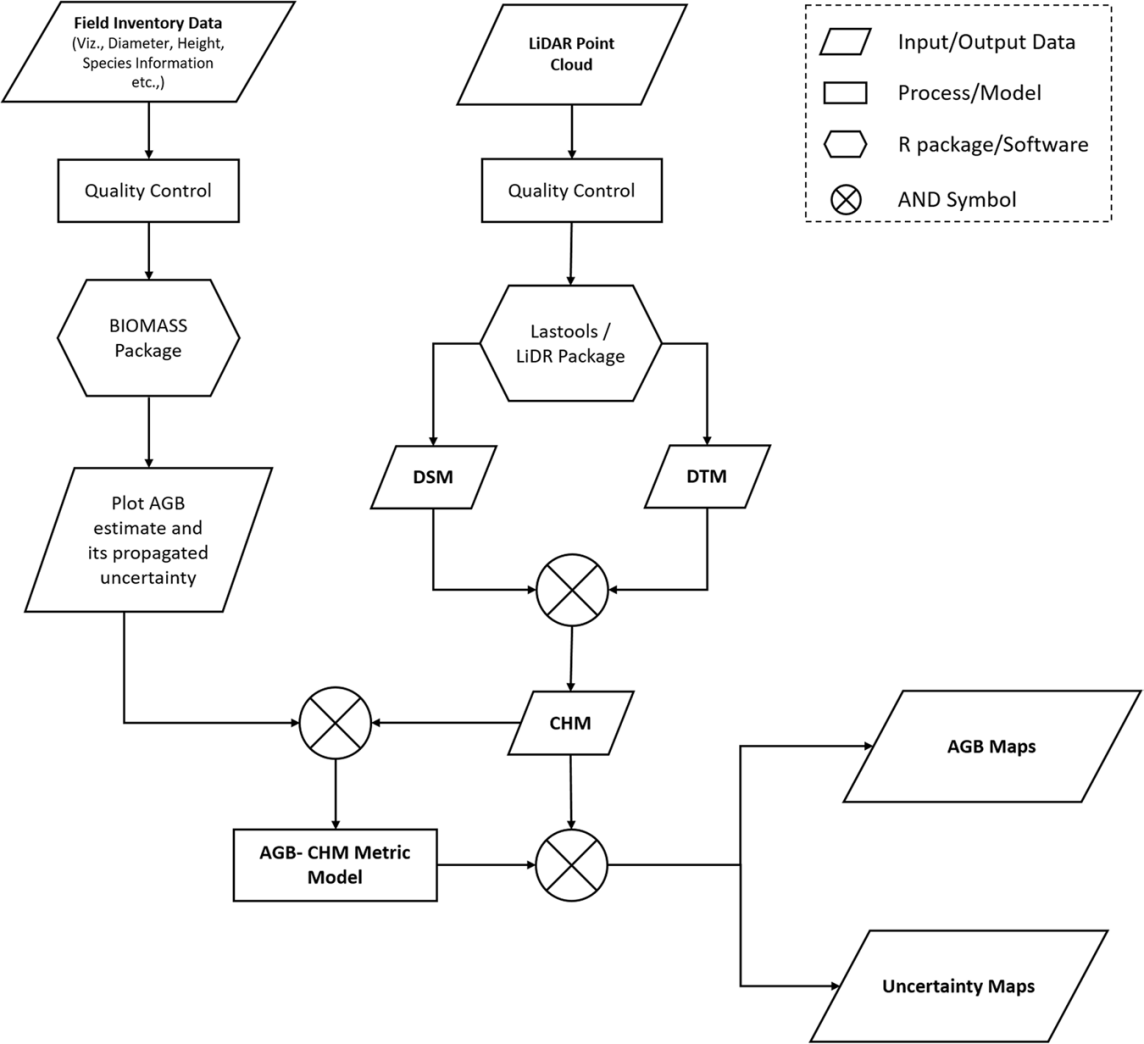
Flow chart depicting workflow of the data analysis procedure to generate reference AGB datasets.

**Fig. 3:**
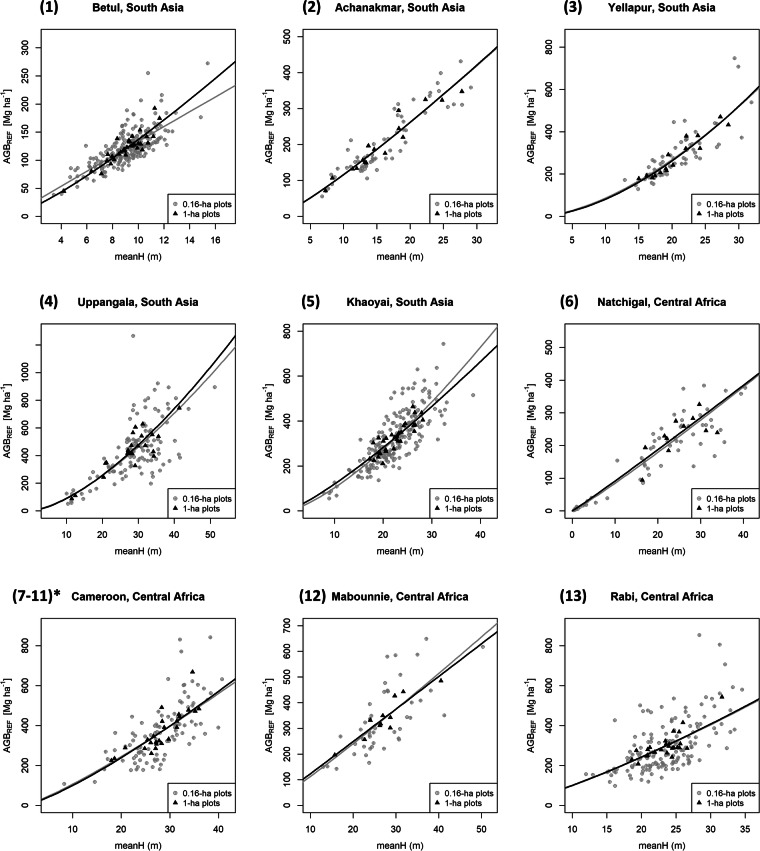
LiDAR-AGB models of Asian and African sites at 1-ha and 0.16-ha resolutions. The numbers for each site refer to Table 1. (7–11)* refers to the regional model established over moist dense forests of Cameroon.

In this context, we aim to generate reference biomass datasets over the tropics for eight sites in Central Africa and five sites in South Asia (Fig. 1) by calibrating airborne LiDAR data with locally established field plots. This paper briefly describes (1) the details of the study sites and the datasets used, (2) the methodology used to generate the reference AGB maps (Fig. 2), and (3) the Monte Carlo simulation workflow used to generate uncertainty maps along with each reference AGB map. Finally, this paper provides access to these reference AGB datasets generated at 100 m and 40 m spatial resolutions over airborne LiDAR footprints ranging from 100 to 40,000 ha.

## Methods

### Sampling sites and associated inventory and LiDAR datasets

We compiled co-located forest inventory and LiDAR datasets from 13 sampling sites in Central Africa and South Asia encompassing an array of abiotic conditions, forest types and structures (Fig. 1; Tables 1 and 2). Forest inventories were carried out at each site, and LiDAR datasets were obtained with an absolute temporal difference of 2.2 ± 1.9 years (range: 0–6.2 years) from the field measurements.

**Table 1:** Sampling site details on forest types, inventory statistics and characteristics of the LiDAR acquisitions.

Sno	Site name	Region	PFT	INV_Date_	Area_INV_	N_range_	BA_range_	LiDAR_Date_	LiDAR_Area_	Platform
1	Betul	S-As	DBT, GSW	02-2016 to 03-2016	46	150–437	7.2–20.2	04-2014	10000	Aircraft
2	Achanakmar	S-As	EBT, DBT	12-2017 to 01-2018	16	186–509	10.0–31.3	10-2015	11000	Aircraft
3	Yellapur	S-As	EBT, DBT, GSW	01-2015 & 01-2016	15	140–749	20.3–43.6	11-2017	40000	Aircraft
4	Uppangala	S-As	EBT	03-2013 & 03-2014	23	370–838	10.3–62.4	11-2013	900	Aircraft
5	Khaoyai	S-As	EBT	11-2015 to 04-2018	34.84	394–1015	20.8–38.1	04-2017	4500	Aircraft
6	Nachtigal	C-Af	DBT, GSW	02-2018 to 05-2018	13.16	216–538	23.4–35.0	01/2012	25000	Aircraft
7	Mbalmayo	C-Af	EBT	04-2021 to 07-2021	9	442–596	20.1–32.5	02-2023	400	UAV
8	Atout	C-Af	EBT	07-2017	2	475–574	30.8–32.0	11-2021	150	UAV
9	Kompia	C-Af	EBT	12-2018	2	423–519	29.0–32.3	12-2019	400	UAV
10	Somalomo	C-Af	EBT	09-2022	8	325–508	25.4–33.6	04-2022	800	UAV
11	Bouamir	C-Af	EBT	12-2018	4	362–552	24.9–35.8	12/2018	1000	UAV
12	Mabounié	C-Af	EBT	04-2012 to 10-2012	11	222–492	17.7–31.4	11/2007	16000	Aircraft
13	Rabi	C-Af	EBT	2015	25	392–532	20.5–36.6	10/2012	1000	Aircraft

Area_INV_ indicates the total area of field inventories, LiDAR_Date_ indicates the month and year of acquisition of LiDAR data and LiDAR_Area_ indicates the total area covered by LiDAR data over the site. N_range_ and BA_range_ indicate the range in number of trees and basal area per hectare across the inventoried area, respectively. The associated plant functional types (PFT’s) for each site are derived from the Moderate Resolution Imaging Spectroradiometer (MODIS) Land Cover Type product (MCD12Q1) which follows Land Cover Type 5 Classification Scheme; a similar strategy is adopted by GEDI Mission.

**Table 2:** Environmental conditions across sampling sites.

Site No.	Site name	Region	Elevation	Slope	MAT	MAP
1	Betul	S-As	487 ± 48	7.8 ± 5.7	25.6 ± 0.3	1266 ± 27
2	Achanakmar	S-As	813 ± 165	8.8 ± 6.8	23.3 ± 0.7	1328 ± 42
3	Yellapur	S-As	459 ± 110	8.7 ± 7.8	24.4 ± 0.5	2383 ± 421
4	Uppangala	S-As	377 ± 122	18.6 ± 8.5	25.1 ± 0.5	3789 ± 63
5	Khaoyai	S-As	757 ± 34	6.8 ± 4.2	23.3 ± 0.1	1127 ± 7
6	Nachtigal	C-Af	527 ± 50	4.5 ± 3.4	24.4 ± 0.5	1588 ± 17
7	Mbalmayo	C-Af	662 ± 11	5.2 ± 3.3	23.6 ± 0.1	1706 ± 1
8	Atout	C-Af	715 ± 11	5.2 ± 3.0	23.7 ± 0.1	1572 ± 1
9	Kompia	C-Af	705 ± 20	5.7 ± 3.3	23.5 ± 0.1	1606 ± 2
10	Somalomo	C-Af	656 ± 13	4.7 ± 3.0	23.7 ± 0.1	1602 ± 3
11	Bouamir	C-Af	696 ± 14	5.3 ± 3.1	23.5 ± 0.1	1616 ± 2
12	Mabounié	C-Af	88 ± 31	7.3 ± 4.6	26.1 ± 0.1	2034 ± 16
13	Rabi	C-Af	68 ± 15	6.3 ± 3.8	25.6 ± 0.1	1826 ± 4

Over the LiDAR acquisition area, the statistics (mean ± standard deviation) of elevation and slope are computed using SRTM at 30-m spatial resolution (V3 product), Mean Annual Temperature (MAT) and Mean Annual Precipitation (MAP) are computed using WorldClim Version 2.1 data.

Forest inventories were conducted by different teams but followed similar protocols. In each plot, the diameter at breast height (DBH or referred to as D in this study, with D ≥ 10 cm) and the taxonomic identification of each tree were recorded. Tree relative coordinates within the plots were measured either at the individual or at the 20 × 20 m quadrat level. For a subsample of trees within the plots, tree height (H) was measured using a laser rangefinder device. Finally, plot geographic coordinates were determined using points measured every 20 m along the plot borders using a combination of differential GPS measurement system and electronic total station (in Asia) or a regular GPS system (in Africa), to warrant an accurate link between ground and remote-sensing data. The complete inventory dataset includes information on D and H measurements for respectively 97,251 and 13,303 trees, and identification rates of 89% at the species level and 92% at the genus level (8% of the trees were left unidentified). The number, size and layout of the inventory plots are uneven across sampling sites with, e.g., a single large 25-ha plot in the Forest-Geo “Rabi” site, a large 30-ha plot and smaller plots of 1-ha and 0.48-ha in the “Khao Yai” site, or a varying number of scattered 1-ha plots (ranging from 2 to 16 in the “Atout” and “Achanakmar” site, respectively). In general, the inventoried extent per site is smaller in Africa (9 ± 8 hectares) than in Asia (27 ± 13 hectares). For a breakdown of plot number, size, tree measurements and identification rates per sampling site, please refer to Table 3.

**Table 3:** Site-level details on field plot layout description, the number of compiled plots at 1-ha (N_1ha_) and 0.16-ha (N_0.16ha_), number of total trees across all plots (N_Trees_), number of trees measured for height (N_Tree_hts_).

Sno	Site	Field Plots description	N_1ha_	N_0.16ha_	N_Trees_	N_Tree_hts_	Species [%]	Genus [%]	Family [%]
1	Betul	Single plot of 34-ha and 12 distributed plots of 1-ha	34	227	15672	677	95%	96%	96%
2	Achanakmar	16 distributed plots of 1-ha	16	64	5750	1546	88%	88%	88%
3	Yellapur	15 distributed plots of 1-ha	15	60	8519	4932	97%	97%	97%
4	Uppangala	Single plot of 10-ha and 13 distributed plots of 1-ha	23	112	14967	1729	61%	61%	61%
5	Khaoyai	Single plot of 30-ha, 1 plot of 1-ha and 8 distributed plots of 0.48 ha	31	192	19461	517	100%	100%	100%
6	Nachtigal	11 distributed plots of 1-ha & 18 distributed plots of 0.16 ha	11	62	4315	703	86%	96%	97%
7	Mbalmayo	9 distributed plots of 1-ha	9	36	4378	532	74%	97%	99%
8	Atout	2 distributed plots of 1-ha	2	8	1049		96%	99%	100%
9	Kompia	2 distributed plots of 1-ha	2	8	942	49	95%	98%	98%
10	Somalomo	8 distributed plots of 1-ha	8	32	4564	215	93%	99%	99%
11	Bouamir	4 distributed plots of 1-ha	4	16	1784	171	92%	98%	98%
12	Mabounié	11 distributed plots of 1-ha	11	44	4425	570	93%	100%	100%
13	Rabi	Single plot of 25-ha	25	144	11425	1662	94%	100%	100%

Species, Genus and Family (%) stands for the identification rate (in %) at the given taxomonic level.

The LiDAR data at each sampling site were acquired between 2012 and 2022 using either aircraft or unmanned aerial vehicles (UAV, Table 1).

### Inventory data processing: computation of reference AGB predictions

Forest inventories were first split into 1-ha (i.e. 100 × 100 m) and 0.16-ha (i.e. 40 × 40 m) plots, using information on tree location recorded in the field (i.e. either individual tree location or quadrat number). The two plot sizes correspond to the two mapping resolutions considered in this study. The 40-m resolution was chosen to account for plots where individual tree locations were only recorded at 20 × 20 m quadrat-level. In cases where the original plot size was not a multiple of the desired output size (typically when splitting 100 × 100 m plots into 40 × 40 m plots), subplots of the desired outputs size were selected at the edges of the original plot, thus leaving-out parts of the original inventory dataset (20 m wide bands in the center as per the previous example). The resulting number of 1-ha and 0.16-ha plots compiled at each sampling site is provided in Table 3.

Subsequently, the BIOMASS R package^18^ (version 2.1.8) within the R statistical platform (version 4.1.3) was used to compute reference AGB predictions for forest inventory plots at the two spatial resolutions (1-ha and 0.16-ha). To that end, we differentiated sites with a cumulated forest inventory area of 10 ha or more (i.e., 8 out of 13 sites, Tables 1 and 3) from those with less than 10 ha of cumulated forest inventory area (i.e., 5 sites). In the former case, we developed site-specific tree height-diameter (H-D) allometric models using second-order polynomials on log-transformed data (modelHD function in the BIOMASS package) and these models were used to predict the height of trees without H measurements in each respective site. In the latter case, which pertained to sites located in moist dense forests of Cameroon (SiteIDs 7 to 11 in Table 1), all inventory data from that country and biome were pooled into a single training dataset and the same H-D modelling procedure was applied. The resulting country- and biome-specific model was then used for predicting tree height at those sites. The H-D model coefficients for these site-level and Cameroon level model are presented in Table 4. Next, a wood density (WD) estimate was attributed to each tree based on its taxonomic identification using the getWoodDensity function.

**Table 4:** H-D Model coefficients (a, b, c) of the 2^nd^ order log-log polynomial model form (${\rm{ln}}\left(H\right)=a+b\times \left({\rm{ln}}\left(D\right)\right)+c\times {\rm{ln}}\left({D}^{2}\right)+\varepsilon$), where H is the height of the tree and D is the tree diameter. *ε* is the normally distributed error to be used during back-transformation for Baskerville correction.

Sno	Site	a	b	c	sigma [*ε*]	R^2^	RMSE [m]
1	Betul	−1.163	1.857	−0.194	0.148	0.71	2.37
2	Achanakmar	−1.314	1.770	−0.159	0.250	0.63	4.06
3	Yellapur	0.563	0.851	−0.050	0.231	0.60	3.75
4	Uppangala	0.164	1.123	−0.077	0.249	0.67	4.33
5	Khaoyai	1.094	0.479	0.025	0.329	0.66	4.90
6	Nachtigal	−0.446	1.457	−0.127	0.223	0.72	5.06
7	Mbalmayo	−0.081	1.286	−0.104	0.243	0.71	4.61
8	Atout						
9	Kompia						
10	Somalomo						
11	Bouamir						
12	Mabounié	0.283	1.102	−0.083	0.236	0.66	5.47
13	Rabi	1.234	0.614	−0.022	0.224	0.58	4.46

Considering that tree AGB prediction is associated with various sources of uncertainty (including measurement errors of the independent variables such as tree diameter, height, and wood density, as well as prediction errors of the H-D models and the AGB allometric model)^5,14^, we used a Monte Carlo approach for uncertainty propagation. Specifically, we employed the AGBmonteCarlo function of the BIOMASS package^18^, which allows propagating the above-mentioned sources of uncertainty and outputs 1000 tree-level and subsequently plot-level AGB predictions. Tree AGB predictions were made using the pantropical AGB allometric model (i.e., Equation-4 in Chave *et al*.^19^). For each plot, the 1000 AGB predictions were (i) averaged to obtain a reference plot-level AGB density (hereafter AGB_REF_) for the development of LiDAR-AGB models and (ii) used for the propagation of uncertainties to the final AGB maps (see section “mapping forest AGB and prediction uncertainty”).

### LiDAR data processing: computation of canopy height metrics

LiDAR data from African and Asian sites were processed using LAStools (version 201124) and the lidR R package (version 4.0.1), respectively. The same processing chain was applied to generate the canopy metrics in both cases. First, a digital surface model (DSM) free of pits and spikes was generated at a 1-m resolution by interpolating the highest points on a 1-m grid. Second, a ground point classification was performed on the point cloud and a digital terrain model (DTM) was interpolated from ground-points. The canopy height model (CHM) was then derived by subtracting the DTM from the DSM. Finally, the 1-m CHM was used to compute 15 canopy metrics for each plot (Table 5) as candidate predictors of forest AGB.

**Table 5:** List of canopy metrics derived from LiDAR-derived CHMs over forest plots extent.

LiDAR Canopy Metric (LCM)	Description
H40	Percentile of CHM values (ex. H98 for the 98th percentile, in m)
H50	
H60	
H70	
H80	
H90	
H98	
meanTCH or meanH	Mean of CHM values (in m)
sdH	Standard deviation of CHM values (in m)
CV	Coefficient of variation of CHM values (meanTCH divided by sdH)
QMCH	Quadratic mean of CHM values
CCF2	Percentage of CHM values above 2, 5 and 10 m (in %)
CCF5	
CCF10	
rumple	Roughness of CHM surface (rumple_index function in lidR R package)

### Specification of a general AGB model form

While LiDAR-based AGB mapping models were trained at the site or regional level (for some Cameroon sites), to minimise local bias in model predictions^14,20^, we privileged the use of (i) a single AGB model form across all sites to facilitate sites inter-comparison and the subsequent use of AGB predictions for spaceborne products calibration/validation and (ii) a simple, parametric modelling approach, keeping the number of predictors to a minimum to avoid overfitting and multicollinearity issues. To specify the AGB model form, we used linear mixed-effects models to identify the most predictive LiDAR-derived canopy height metrics (LCMs) on AGB_REF_ variation while accounting for the hierarchical spatial structure of the data. In practice, we built 15 linear mixed-effects models (one for each LCM) on the log-transformed variables of AGB_REF_ and LCM (Eq. 1):

1 $$\log \left({AGB}_{REF}\right)=a+b\times \log \left(LCM\right)+{RE}_{site}+\varepsilon $$

where *a* and *b* are the model’s coefficients, LCM represents the Lidar-derived Canopy Metric, AGB_REF_ corresponds to the field-derived AGB prediction at a given spatial resolution (i.e. 0.16- or 1-ha), RE_site_ denotes the random site effect used in linear mixed-effects modelling and *ε* is the error term, assumed to follow a normally distribution with a mean of zero and a standard error σ. Based on the AIC criterion, the meanTCH metric (i.e. the mean of all CHM values in the plot area) emerged as the best predictor of AGB_REF_ variation at both 1-ha and 0.16-ha spatial resolutions (Table 6).

**Table 6:** LiDAR-AGB Linear Mixed Effects Model performance statistics at 1-ha and 0.16-ha plot sizes.

Plot Size (1-ha)					Plot Size (0.16-ha)				
LCM	AIC	R^2^	RMSE [Mg ha^−1^]	RMSE [%]	LCM	AIC	R^2^	RMSE [Mg ha^−1^]	RMSE [%]
meanH	−180.42	0.89	43.62	15.2	meanH	149.94	0.71	88.55	32.4
RH60	−169.22	0.88	44.75	15.6	QMCH_chm	194.11	0.70	89.31	32.7
RH50	−167.22	0.88	45.27	15.8	RH90	379.89	0.65	96.81	35.4
RH70	−151.53	0.87	45.83	16.0	RH98	642.43	0.59	105.29	38.5
QMCH_chm	−148.49	0.88	45.30	15.8	RH40	869.25	0.60	106.49	39.0
RH80	−133.52	0.87	47.14	16.5	RH50	889.42	0.59	106.84	39.1
RH40	−114.58	0.86	48.46	16.9	CCF10	928.45	0.50	116.11	42.5
RH90	−102.74	0.85	50.54	17.7	RH60	975.37	0.59	106.68	39.0
RH98	−23.33	0.79	59.26	20.7	RH80	1033.34	0.62	103.08	37.7
CCF10	15.74	0.72	70.11	24.5	RH70	1036.05	0.59	106.41	38.9
CCF5	66.87	0.63	79.56	27.8	CCF5	1101.04	0.46	120.43	44.1
CV	85.22	0.61	81.18	28.4	CCF2	1105.63	0.45	121.80	44.6
CCF2	90.21	0.60	82.20	28.7	CV	1265.00	0.41	132.29	48.4
sdH	114.75	0.62	79.52	27.8	sdH	1365.98	0.41	128.62	47.1
rumple	131.76	0.58	83.77	29.3	rumple	1473.62	0.39	130.34	47.7

The table is sorted in ascending order based on the column “AIC” (Akaike information criterion) when the respective LiDAR Canopy Metric (LCM) is used for Eq. 1. R^2^ and RMSEs (in Mg ha-1 and in %) are computed on back-transformed predictions.

A similar procedure was run on AGB_REF_ prediction models combining each pair of LCMs rather than a single predictor. At both spatial resolutions, the best two-predictor model resulted in a modest improvement in relative RMSE (i.e., <0.2%, Table 7) compared to the model based on meanTCH only. The latter model form was thus selected for biomass mapping. In line with the H:D modelling procedure, LiDAR-based AGB mapping models were either trained at the site-level (for sites with a cumulated forest inventory area of 10 ha more) or on a pooled training dataset containing all inventory data from Cameroonian moist dense forests (for sites with a cumulated forest inventory area smaller than 10 ha), henceforth referred to as the “regional” AGB model. It is noteworthy that including sites as an additional fixed-effect covariate in the regional model did not yield significant effects for this variable at a 5% risk (neither in terms of site-level intercepts nor in terms of interactions between sites and the meanTCH predictor), suggesting a minimal site effect on the regional model’s predictions, if any.

**Table 7:** LiDAR-AGB Linear Mixed Effects Model performance statistics at 1-ha and 0.16-ha plot sizes using two LCMs as predictive variables.

Plot Size (1-ha)					Plot Size (0.16-ha)				
LCMs	AIC	R^2^	RMSE [Mg ha^−1^]	RMSE [%]	LCMs	AIC	R^2^	RMSE [Mg ha^−1^]	RMSE [%]
RH50, RH90	−192.69	0.89	43.75	15.3	meanH, CCF2	114.03	0.71	88.00	32.2
RH50, RH80	−191.69	0.88	43.84	15.3	RH40, meanH	131.70	0.71	88.09	32.2
RH50, QMCH_chm	−188.18	0.88	43.87	15.3	RH98, QMCH_chm	134.13	0.71	87.66	32.1
RH50, RH70	−188.15	0.88	44.18	15.4	meanH, CCF10	136.58	0.71	88.07	32.2
RH50, meanH	−186.09	0.89	43.67	15.3	RH50, meanH	137.09	0.71	88.22	32.3
RH40, RH80	−185.00	0.88	43.96	15.4	meanH, QMCH_chm	139.23	0.71	88.29	32.3
RH40, RH50	−183.00	0.88	44.67	15.6	RH70, meanH	139.36	0.71	88.44	32.4
RH50, RH60	−182.72	0.88	44.37	15.5	RH60, meanH	139.42	0.71	88.36	32.3
RH40, RH90	−182.32	0.88	44.15	15.4	meanH, CCF5	141.40	0.71	88.14	32.3
RH40, RH70	−181.96	0.88	44.26	15.5	RH90, meanH	147.02	0.71	88.54	32.4

The table is sorted in ascending order based on the column “AIC” (Akaike information criterion) when the respective LiDAR Canopy Metrics (LCM) are used in Eq. 1. R^2^ and RMSEs (in Mg ha^−1^ and in %) are computed on back-transformed predictions.

The coefficients and calibration statistics of LiDAR-based AGB mapping models are provided in Table 8, while Fig. 3 shows scatterplots of ‘reference’ against predicted AGB values.

**Table 8:** Model coefficients along with standard errors (in brackets) for site-wise level models at 1-ha and 0.16-ha resolution.

Sno	Site	Plot Size (1-ha)						Plot Size (0.16-ha)					
		a (se)	b (se)	sigma	R^2^	RMSE [Mg ha^−1^]	RMSE [%]	a (se)	b (se)	sigma	R^2^	RMSE [Mg ha^−1^]	RMSE [%]
**1**	**Betul**	2.043 (0.191)	1.247 (0.087)	0.095	0.87	12.04	9.9	2.605 (0.107)	0.988 (0.049)	0.162	0.65	21.08	17.3
**2**	**Achanakmar**	2.046 (0.219)	1.173 (0.080)	0.113	0.94	23.51	11.7	2.058 (0.175)	1.165 (0.064)	0.185	0.84	36.58	18.3
**3**	**Yellapur**	0.500 (0.478)	1.691 (0.158)	0.111	0.90	31.07	11.0	0.684 (0.380)	1.632 (0.126)	0.188	0.74	66.65	23.1
**4**	**Uppangala**	0.969 (0.442)	1.523 (0.134)	0.192	0.86	82.16	18.9	1.087 (0.335)	1.464 (0.100)	0.328	0.66	145.85	32.6
**5**	**Khaoyai**	1.934 (0.445)	1.236 (0.144)	0.109	0.72	32.27	9.9	1.360 (0.189)	1.411 (0.061)	0.219	0.74	74.08	23.1
**6**	**Nachtigal**	2.009 (1.004)	1.037 (0.314)	0.233	0.55	43.21	18.6	1.902 (0.081)	1.083 (0.029)	0.284	0.82	48.28	27.9
**7**	**Mbalmayo**	1.721 (0.517)	1.253 (0.155)	0.135	0.74	52.62	14.1	1.230 (0.396)	1.39 (0.119)	0.282	0.59	108.54	29.1
**8**	**Atout**												
**9**	**Kompia**												
**10**	**Somalomo**												
**11**	**Bouamir**												
**12**	**Mabounié**	2.471 (0.447)	1.015 (0.136)	0.100	0.86	32.26	9.4	2.159 (0.498)	1.098 (0.152)	0.265	0.55	94.23	28.0
**13**	**Rabi**	1.267 (0.671)	1.397 (0.213)	0.123	0.65	38.78	13.1	1.386 (0.407)	1.344 (0.130)	0.312	0.43	99.63	33.8

For Cameroon sites listed from 7-11 in column “Sno”, a single regional model is employed. Sigma is the model residual standard error in log-transformed units. R^2^ and RMSEs (in Mg ha-1 and in %) are computed on back-transformed predictions.

### Mapping forest AGB and prediction uncertainty

We mapped forest AGB and prediction uncertainty over the extent of airborne LiDAR data at each site using a Monte Carlo approach similar to that used to compute plot-level AGB_REF_. More specifically, we used the 1000 plot-level AGB predictions generated at the first modelling level (i.e., from tree to plot) to build 1000 LiDAR-based models per site (or at “regional” level for Cameroonian sites with less than 10 ha of cumulated forest inventory area). At the second modelling level (i.e., from plot to landscape), pixel AGB predictions derived from LiDAR-based models suffer from additional uncertainty associated to the LiDAR-based models themselves. To propagate this additional uncertainty, we mimicked the procedure used in BIOMASS to propagate the uncertainty associated to the tree-level AGB allometric model (see Appendix S1 of Réjou-Méchain *et al*.^18^ for codes and details), which entailed using a Markov chain Monte Carlo algorithm to infer the uncertainty on Lidar-based models’ parameters (i.e., models’ coefficients and associated RSE). The Markov chain outputted 1000 sets of model parameters per model. For each of the 1000 LiDAR-based model at each site, we then (1) randomly selected a set of parameters among the 1000 available sets, (2) used the model coefficient selected in (1) to predict pixels AGB and (3) added to all pixels an error term randomly drawn from a normal distribution *N*(0, RSEi) where RSEi is the model RSE selected in (1). This procedure led to 1000 predictions of pixels AGB embedding the prediction uncertainty from both the first and second modelling levels. Finally, reference AGB maps and associated spatial uncertainty maps were generated as the mean and standard deviation of the 1000 pixel AGB predictions, respectively. Hereafter, we refer to pixels mean AGB prediction as AGB_PRED_.

### Additional metadata for the AGB maps

LiDAR-based AGB maps produced in the present study are intended to support calibration and validation efforts of spaceborne data. To maximise their usefulness, we provide additional information that users may require – depending on their study’s objective and methodological choices – to facilitate their integration with spaceborne data and/or develop comprehensive uncertainty propagation schemes up to the final, spaceborne-derived AGB map.

A first challenge users may face relates to the computation of the uncertainty associated with the mean AGB of arbitrary subregions of LiDAR AGB maps. Such subregions could for instance correspond to the footprints of spaceborne data unit pixels. Estimating the total mean squared error associated with a map (sub) population mean requires access to the matrix of pairwise population unit covariances, which is rarely communicated by map makers to users because of its large size. Yet, McRoberts *et al*.^21^ recently showed that pairwise population unit covariances could largely contribute to total mean squared error, and proposed an averaging and binning approach to drastically reduce the matrix size, thus facilitating its publication along with AGB maps. While we refer interested readers to McRoberts *et al*.^21^ for methodological details, we provide in Supplementary data all information recommended by the authors to allow map users to comply with IPCC good practice guidelines for greenhouse gas inventories. We note that for each pixel of the LiDAR-based AGB maps provided in this study, a bin number is available in the third map layer.

Another challenge lies in the propagation of uncertainties in multi-level hierarchical modelling, which is a likely use-case of the LiDAR-based maps we produced. These maps were generated by applying two hierarchically nested models: a tree allometric model linking field measurements to tree AGB, and a mapping model linking plot AGB to LiDAR data. LiDAR-based AGB maps users may employ a three-steps hierarchical modelling approach and add as a third step a model linking high resolution AGB predictions from the LiDAR-based maps to the coarser resolution of spaceborne data. An example of such an approach is presented in detail in Saarela *et al*.^22^ and referred to as “three-phase hierarchical model-based inference”. The uncertainty assessment in such a nested modelling approach requires information at the two first modelling steps that goes beyond the results of the Monte Carlo simulation we used to produce pixel-level uncertainty estimates. While we refer interested readers to Saarela *et al*.^22^ for methodological details, we provide in Supplementary data all information allowing users to assess uncertainty as described in Saarela *et al*.^22^. This information notably includes the variance-covariance matrix of model parameters for each sampling site as well as statistics on parameters (DBH, AGB, pixels’ height from CHM, etc.,) used at various levels in the chain of hierarchical models.

## Data Records

For each site, AGB and uncertainty maps are distributed as a single GeoTiff file at the two spatial resolutions (1- and 0.16-ha) through Dataverse^23^. Each file comprises three individual layers. The two first layers named meanAGB and sdAGB correspond to the mean and standard deviation of AGB predictions over the 1000 Monte Carlo simulations, respectively. The file projection system is Universal Transverse Mercator. The third layer named *Nbin* corresponds to the bin number each map pixel is associated with in the binning approach proposed by McRoberts *et al*.^21^ to allow users reconstituting a matrix of pairwise population unit covariances estimates.

Besides data access through Rodda *et al*.^23^, data from Asian sites can be access and visualized through the Bhuvan Portal (https://bhuvan-app3.nrsc.gov.in/data/download/index.php). To access the visualisation/download through Bhuvan Portal, select the ISRO Geosphere-Biosphere Programme under the Program option and then choose the group Above Ground Biomass (AGB) Data.

In addition, we provide two supplementary data files (in excel format) that provide additional metadata details on site-level binned covariance matrices and variance-covariance matrices and summary of all the parameters used in the present study.

## Technical Validation

**Fig. 4:**
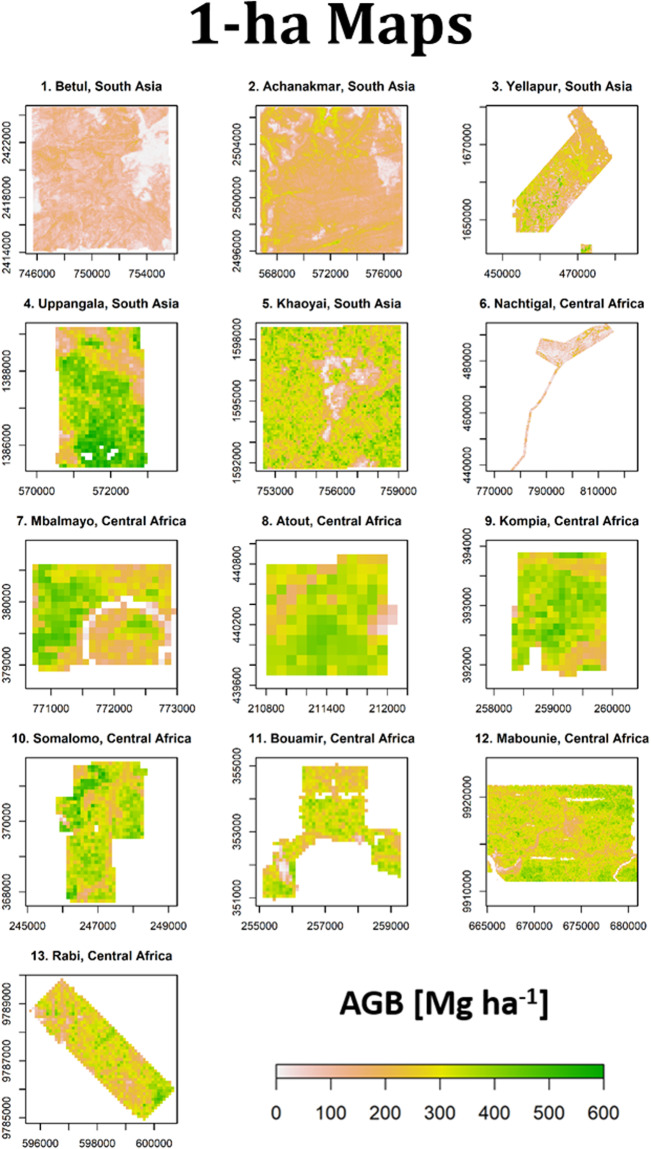
Reference AGB maps of Asian and African sites at 1-ha spatial resolution.

**Fig. 5:**
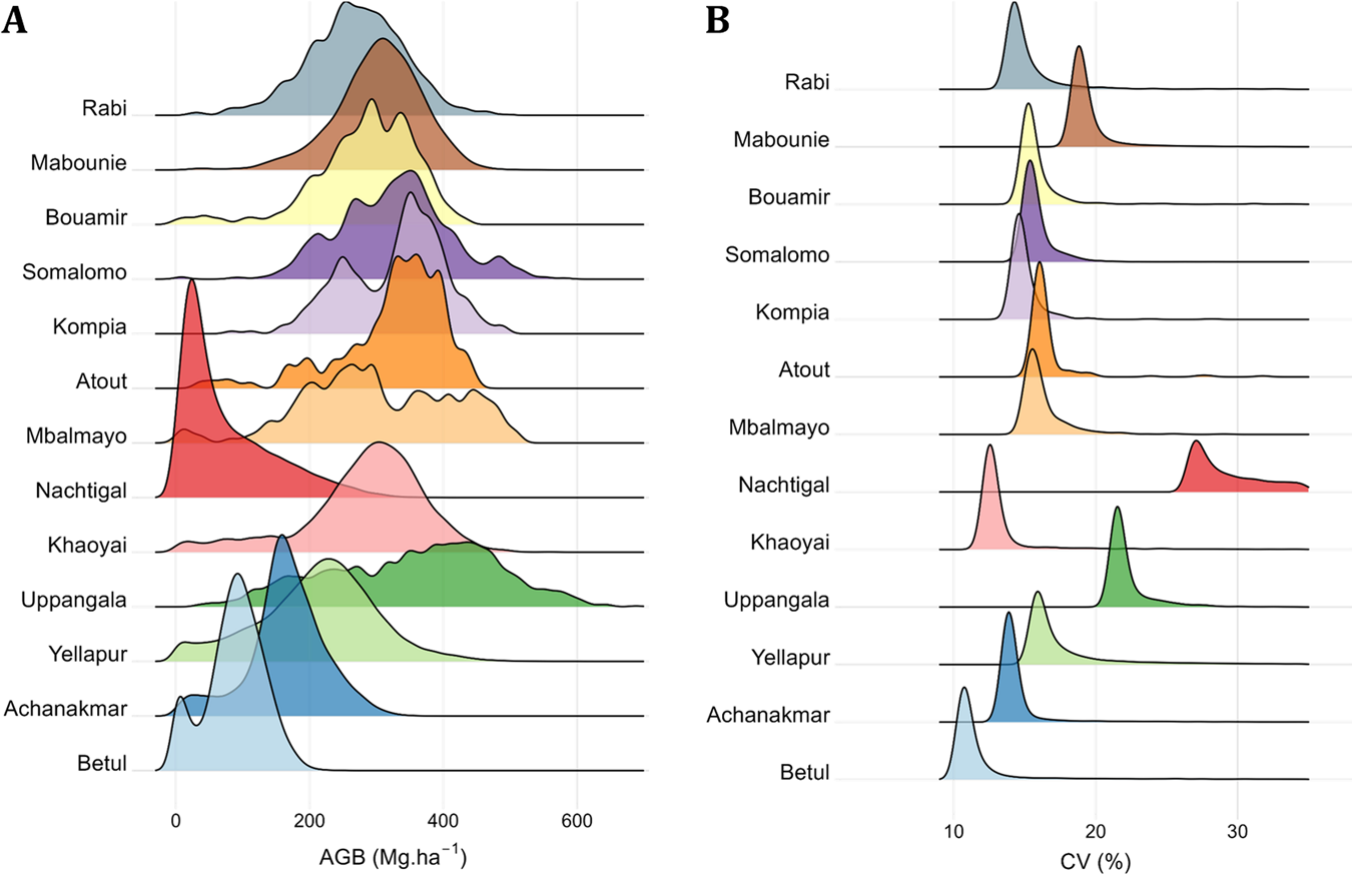
Density distributions of (**A**) mean pixel AGB and (**B**) AGB uncertainty, expressed as a coefficient of variation (CV, in %), at 1-ha mapping resolution across sites.

**Fig. 6:**
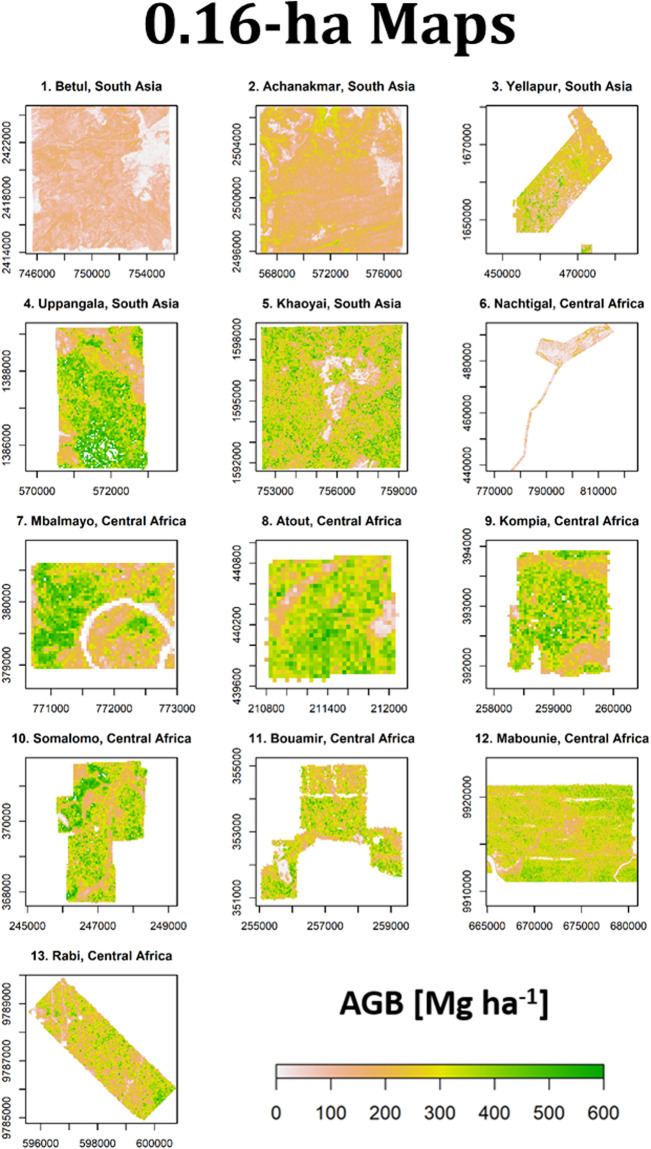
Reference AGB maps of Asian and African sites at 0.16-ha spatial resolution.

**Fig. 7:**
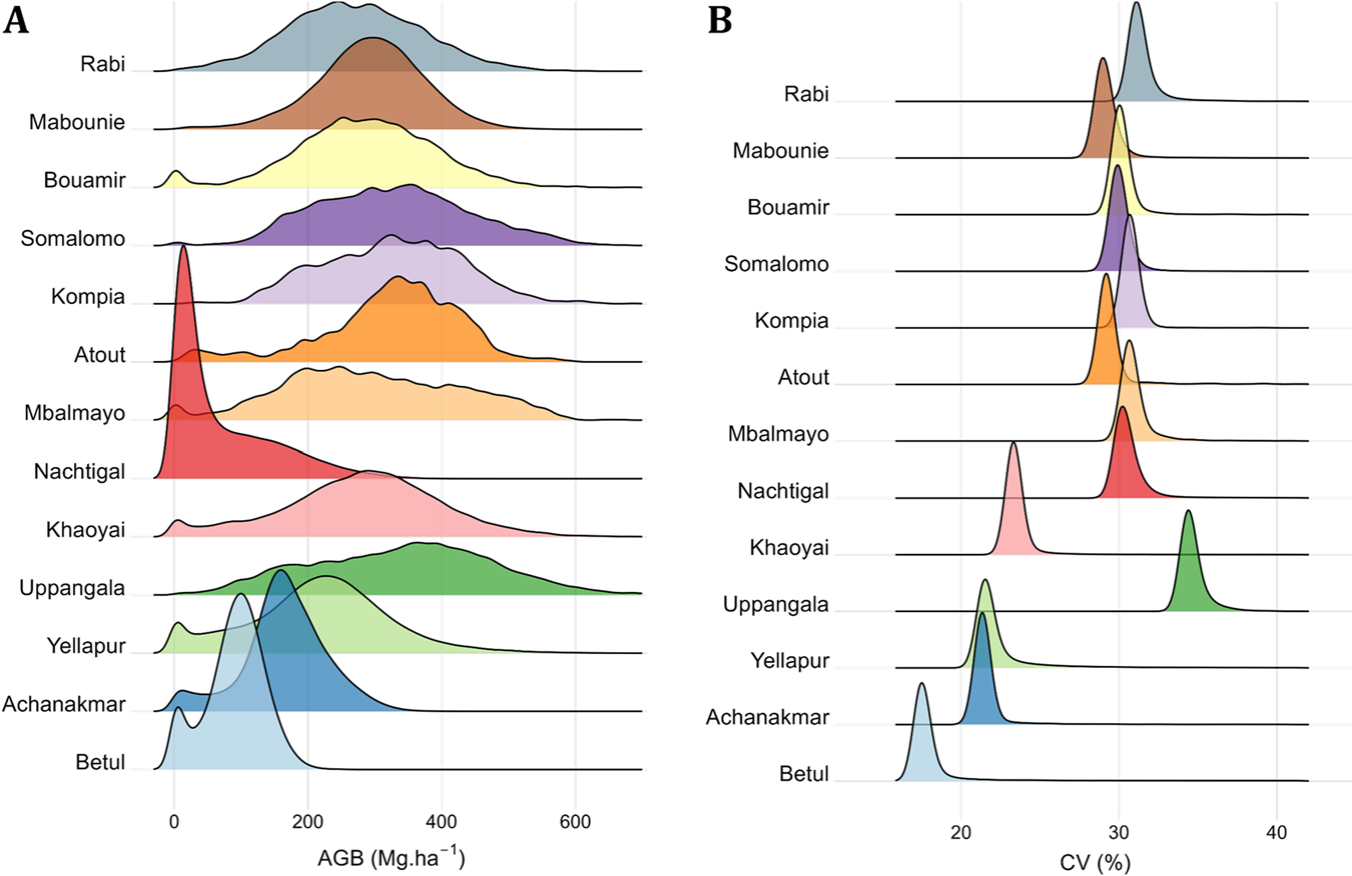
Density distributions of (**A**) mean pixel AGB and (**B**) AGB uncertainty, expressed as a coefficient of variation (CV, in %), at 0.16-ha mapping resolution across sites.

Reference AGB maps at 1-ha resolution are shown in Fig. 4 and the density distributions of 1-ha AGB maps are represented in Fig. 5-A along with uncertainty levels in Fig. 5B expressed as a coefficient of variation (CV, in % of mean AGB) (see Fig. 6 and Fig. 7 for AGB maps and respective density distributions at 0.16-ha resolution). Figure 5-B shows that the mean uncertainty across sites is 15.4%, with site-level mean uncertainty ranging from 10.8 to 31%. It can be observed that Nachtigal and Uppangala sites have larger mean uncertainties than other sites, with 31% and 20.1%, respectively. This can be explained by larger LiDAR-AGB model uncertainties at these sites and mapping resolution (see models’ “sigma” in Table 8).

In addition to the per pixel estimates of uncertainty accompanying AGB maps, we hereafter provide (i) an assessment of mapping model predictive performances using a spatial model cross-validation technique^24^, to provide additional insights into the reliability of AGB predictions on each map and (ii) an assessment of mapping models extrapolation at each sampling site, which may be useful to help users for filtering-out pixels where extrapolation occurred and only retaining the highest quality AGB predictions for spaceborne products calibration/validation.

### Model spatial cross-validation

Model calibration statistics in Table 8 likely overestimate model predictive performance on pixels that are not used for model training, that is, on most maps’ pixels. We performed a cross-validation (CV) of each model to provide more reliable insights into model predictive performance. Field plots at each site are iteratively split into training and test data and model CV statistics are built on the set of test data predictions. Regarding CV design, we selected a buffered leave-one-out cross-validation (LOO-CV^25^) where a spatial buffer around test data is used to exclude from model training dataset observations located at the neighbourhood of test data, thus avoiding inflation in CV statistics due to spatial autocorrelation in forest AGB^24^. As a compromise between the diversity in terms of number and spatial arrangement of field data across sites (e.g. multiple individual 1-ha plots vs. single large plot), the consistency of the CV approach across sites, as well as our expectation for a relatively weak spatial autocorrelation in forest AGB at the high resolution of the maps (<100 m^2^)^26^, we selected a LOO-CV with a 100 m buffer radius for all sites and mapping resolutions (i.e. 100 × 100 m and 40 × 40 m). This CV design notably implies that (i) when a test observation came from a large field plot (i.e. >1-ha, e.g. the 25-ha plot at Rabi), subplots at its direct neighbourhood were not used for model training (i.e., all subplots intersecting a 100 m circular buffer around the center of a test subplot were excluded from the training set, regardless of the mapping resolution), and (ii) at the 40 × 40 m mapping resolution, when a test observation came from a 1-ha field plot, the remaining three subplots of that 1-ha plot were not used for model training. The results of the buffered LOO-CV are presented in Table 9. They show that the predictive performances of mapping models developed in this study are comparable to those found in the literature (i.e. 15–20% on average for the tropical forest biome^15^) with relative RMSEs ranging from 10.6 to 20.1% (mean across sites: 14.1%) at 1-ha and 17.7 to 33.7% (mean across sites: 25.7%) at 0.16-ha.

**Table 9:** Error statistics of modified LOO-CV procedure at site-level for 1-ha and 0.16-ha plots.

Sno	Site	Plot Size (1-ha)			Plot Size (0.16-ha)		
		R^2^	LOOCV-RMSE [Mg ha^−1^]	LOOCV-RMSE [%]	R^2^	LOOCV-RMSE [Mg ha^−1^]	LOOCV-RMSE [%]
1	Betul	0.76	12.85	10.5	0.55	21.47	17.6
2	Achanakmar	0.90	27.05	13.5	0.81	39.00	19.5
3	Yellapur	0.85	36.27	12.9	0.62	74.21	25.8
4	Uppangala	0.64	100.44	23.2	0.37	171.68	38.4
5	Khaoyai	0.65	37.40	11.5	0.62	75.05	23.4
6	Nachtigal	0.22	60.49	26.1	0.81	49.85	28.8
7	Mbalmayo	0.66	56.36	15.1	0.46	110.39	29.6
8	Atout						
9	Kompia						
10	Somalomo						
11	Bouamir						
12	Mabounié	0.79	37.47	11.0	0.46	96.32	28.7
13	Rabi	0.54	46.11	15.5	0.39	101.74	34.5

### Model extrapolation in the predictor space

**Fig. 8:**
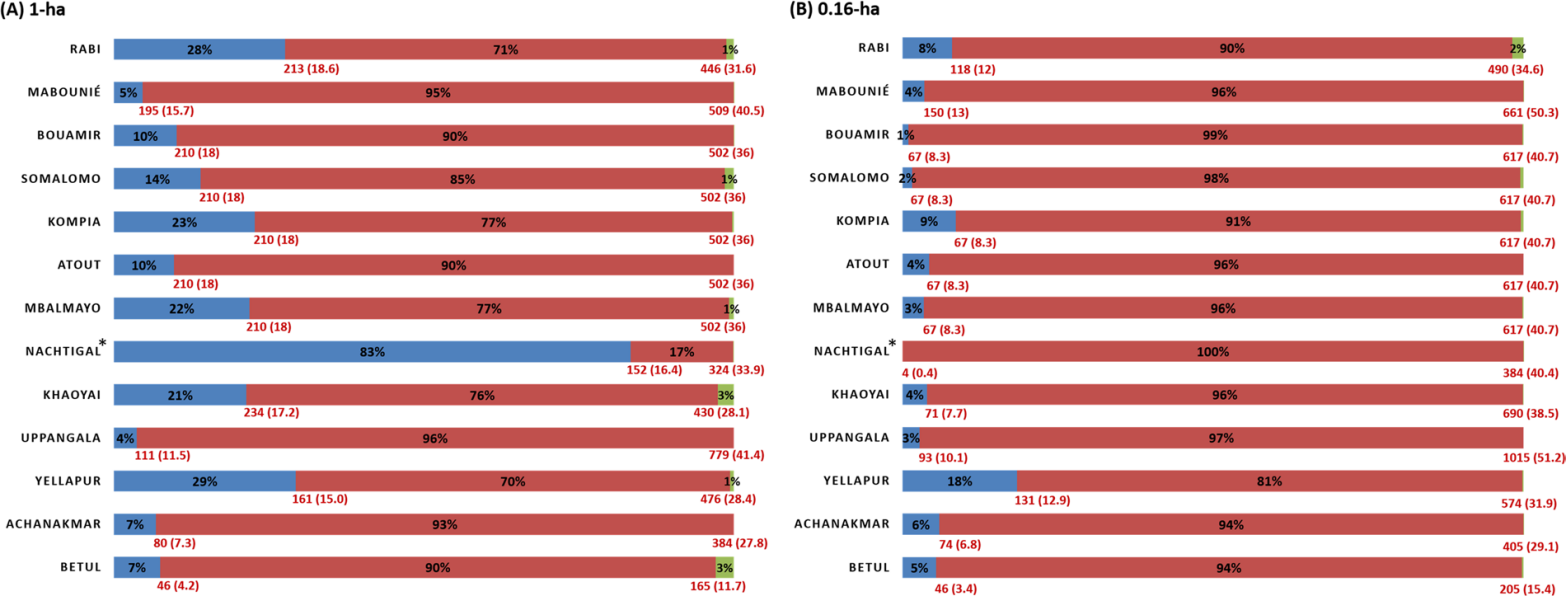
Proportion (in %) of map pixels outside and inside models calibration domains at 1-ha (panel **A**) and 0.16-ha (panel **B**) mapping resolutions. The proportions are computed with respect to the total number of map pixels with CHM > 2 m at the exception of the Natchigal site where a 0.4 m threshold is used so as to account for the nature of the site i.e., a forest-savanna mosaic. The proportion of map pixels within model calibration domains is represented in red. Map pixels below and above the range of model calibration domains are represented in blue and green, respectively.

Uncertainty maps, AGB maps, and model CV results provide insights into the reliability of AGB predictions within the calibration domain of mapping models. It is however likely that the entire gradient of forest structure sampled by LiDAR data was not fully sampled in the model’s training set, thus leading to situations of predictive extrapolation where prediction uncertainty is unknown. To investigate this issue, we compared the range of vegetation height (i.e., meanTCH) sampled by the training set of each mapping model to the full range found in the LiDAR data, restricting the analysis to pixels considered as vegetated, i.e., with meanTCH ≥ 2 m. We found that the proportion of pixels affected by predictive extrapolation strongly varied across sites and at the two mapping resolutions. Generally, the upper range of meanTCH (and thus of AGB_PRED_) found at a landscape scale in the LiDAR data were sampled in the training set (Fig. 8A,B), which probably is a reflection of the “majestic forest bias”^27^ – that is, the tendency for researchers to preferentially establish sample plots where forest stands appear the less disturbed (e.g. tallest canopy height, the highest abundance of large trees, etc.). However, a varying and often substantial proportion of maps on the lower end of the meanTCH gradient was outside the model’s calibration domain. For instance, in the Nachtigal site predictive extrapolation occurred on about 83% of the vegetated pixels on the 1-ha AGB map. This can be explained by the nature of this site, a forest-savanna mosaic, where the meanTCH of all herbaceous and shrubby savannas is lower than the height of the smallest 1-ha forest stand (ie., 16.4 m) found in model training set (Fig. 8A). However, this proportion dropped to 0% at the 0.16-ha mapping resolution thanks to the inclusion into the model training set of 18 additional 0.16-ha plots established in savannas-dominated areas (Fig. 8B, Table 3).

We thus advise potential users of the AGB maps published here to carefully consider the bounds of the mapping model calibration domain at each site (for which we provided corresponding AGB_PRED_ values in Fig. 8) when using these maps as reference data for larger-scale product calibration/validation.

## Usage Notes

Forest AGB maps released here constitute reference estimations for the community of remote-sensing scientists interested in forest carbon stocks. For instance, we expect these maps to be of utmost usefulness for the calibration and validation of next-generation broad-scale aboveground biomass mapping models based on data from ongoing or upcoming spaceborne missions (viz., NASA”s GEDI, NASA-ISRO’s NISAR and ESA’s BIOMASS missions). These data can also be useful when assessing the accuracy of existing maps or recalibrating them (as in eg.^28,29^), especially since study sites presented here are located on renowned data-poor regions^16^ and are marked by notable uncertainties in AGB estimates^17^. That said, we encourage users to account for the dates of LiDAR and ground data acquisitions underlying reference AGB estimates (cf. Table 1), as temporal discrepancies with spaceborne signals or products should ideally be accounted for in any calibration or validation exercise.

More broadly, our study highlights sites of potential interest to build a network of “super-sites” (*sensu*^11^) across the tropics, that is sites combining forest inventory data over sizable areas (≥10 ha) – ideally featuring multiple forest censuses – with airborne LiDAR data. Such data have been collected thanks to the long-term vision of few organisations, to dedicated experts and to the efforts of trained labour forces in the past decades. Ongoing global changes makes the sustained monitoring of permanent forest plots in long-term study sites critical, so as to allow measuring their impacts on forest ecosystems. In spite of this crucial stake, access to funding in the tropical world for replacement of expertise, training programs and to support field data acquisition campaigns is critically limited. We thus urge National and International research and space agencies to ensure long-term funding for on-ground forest research in the tropics.

### Supplementary information

Binned covariance matrices

Variance Covariance Matrices and Parameter Summary

## Data Availability

All statistical analyses were performed in R (v.4.1.3). The BIOMASS R-package is an open source library available from the CRAN R repository. The BIOMASS vignettes and individual function helps contain detailed notes on usage to derive plot level AGB estimates with uncertainty estimates through error propagation using Monte Carlo method. The codes associated with the error propagation from plot-scale AGB to LiDAR AGB maps, generating additional metadata and binned covariance matrices as described in Methods section are available on GitHub (https://github.com/surajreddyr/LIDAR_AGB/tree/main).
